# Bromate and trace metal levels in bread loaves from outlets within Ile-Ife Metropolis, Southwestern Nigeria

**DOI:** 10.1016/j.toxrep.2014.05.007

**Published:** 2014-05-22

**Authors:** J.A.O. Oyekunle, A.S. Adekunle, A.O. Ogunfowokan, G.O. Olutona, O.B. Omolere

**Affiliations:** a Department of Chemistry, Obafemi Awolowo University, Ile-Ife, Nigeria; b Department of Chemistry and Industrial Chemistry, Bowen University, Iwo, Nigeria

**Keywords:** Bread, Bromate levels, Trace metals, Ile-Ife, Nigeria

## Abstract

Bread loaves randomly sampled from nine outlets and bakeries within Ile-Ife were analysed to determine their safety levels for human consumption with respect to bromate and trace metal contents. Bromate determination was carried out *via* spectrophotometric method while trace metals in the digested bread samples were profiled using Flame Atomic Absorption Spectrophotometer. Bromate levels in the analyzed bread samples ranged from 2.051 ± 0.011 μg/g to 66.224 ± 0.014 μg/g while the trace metal levels were of the order: 0.03–0.10 μg/g Co = 0.03–0.10 μg/g Pb < 0.23–0.46 μg/g Cu < 2.23–6.63 μg/g Zn < 25.83–75.53 μg/g Mn. This study revealed that many bread bakers around Ile-Ife had not fully complied with the bromate-free rule stipulated by NAFDAC contrary to the “bromate free” inscribed on the labels of the bread. The bread samples contained both essential and toxic trace metals to levels that could threaten the health of consumers over prolonged regular consumption.

## Highlights

• This study evaluates the levels of bromate and trace metals in the bread loaves sold within Ile-Ife, Nigeria.
• Bromate was determine *via* spectrophotometric method while trace metals were profiled using Atomic Absorption Spectrophotometer.
• Study revealed that many bread bakers had not fully complied with the bromate-free rule stipulated by government contrary to “bromate free” inscribed on the labels of the bread.
• The bread samples contained both essential and toxic trace metals to levels that could threaten the health of consumers over prolonged regular consumption.

## 1 Introduction

Bread is an important staple food in many countries of the world especially the African countries and South Eastern part of Asia [1], [2]. Statistical analysis in Nigeria showed that bread is one of the most consumed food types in homes, restaurants and hotels with predominant consumption among the poor [3] and young ones who constitute more than 70% of the over 150 million people in Nigerian.

Bread is made from low protein wheat flour. Some of the basic ingredients, apart from flour, are table salt, sugars, flavours, and at least, a flour improver such as potassium bromate [4], [5], [6]. Potassium bromate as a flour improver has been in use for more than 80 years [5], [7], [8]. The use of potassium bromate has been a common choice among flour miller and bakers throughout the world because it is cheap and probably the most efficient oxidizing agent [9]. It acts principally as a maturing agent in the late dough stage giving strength to the dough during the late proofing and early baking [5]. It acts as a slow oxidizing agent throughout the fermentation proofing and baking process affecting the structure and the rheological properties of the dough. It is believed to act by oxidizing thiol groups to disulphide linkages, thus strengthening the protein network [10]. It helps bread to rise in the oven and to create a good texture. This property has been manipulated by many Nigerian bakers in profit making.

Toxicological studies have shown that, with respect to human health, consumption of potassium bromate can lead to non-cancer effects, such as degrading vitamins A1, A2, B1, B2, E and niacin which are the main vitamins available in bread [11], [12]; causing significant differences in essential fatty acid content of flour treated with bromate [13]; development of cough, sore throat, abdominal pain, diarrhoea, nausea, vomiting, kidney failure, hearing impairment, bronchial and ocular problems, haemolysis, extreme irritation and injury to tissues especially those of the central nervous system and kidneys when inhaled [14], [15]. On the other hand, numerous studies [16], [17], [18], [19] have indicated that potassium bromate has the potential to cause cancer in both experimental animals and humans by inducing oxidative stress in tissues [12], [20], [21], [22], [23], [24].

The International Agency for Research on Cancer (IARC) has classified potassium bromate as a class 2B carcinogen (a possible human carcinogen) based on sufficient evidence that potassium bromate induces cancer in experimental animals [25], [26]. This led to the proposal for its ban in the United States and several other countries including the United Kingdom in 1990, Canada in 1994, Sri Lanka in 2001 and China in 2005. The FDA and China permit the use of potassium bromate up to a maximum level in bread of 50 mg/kg of flour mass, but Japan permits its inclusion only up to 10 mg/kg of flour [27]. In California a warning label is required when bromated flour is used and currently, it is recognized that it is inappropriate to use potassium bromate in any product or production method which cannot be formulated without residues below the level of 20 ppb (*i.e.* 0.020 mg/kg or 0.020 μg/g) in the finished product [28]. Joint FAO/WHO [11] committee's initial recommendation of acceptable level of 0–60 mg KBrO_3_/kg flour was withdrawn because long term toxicity and carcinogenicity studies *in vitro* and *in vivo* revealed renal cell tumours in hamsters.

The use of potassium bromate in flour milling and baking was banned in Nigeria by National Agency for Food, Drug Administration and Control (NAFDAC) in 2003, and its use infringes on the drug and related products registration decree 20 of 1999 and NAFDAC Decree 15 of 1993 [29]. However, since the ban, it is not certain to what extent Nigerian bakers have complied with the ban imposed by NAFDAC with respect to the use of potassium bromate in bread baking.

Apart from the bromate content, materials employed in bread making and the environments where these bakeries are located are not free from varying degrees of trace metal contaminations. Trace metal contamination could be from the raw materials employed in bread baking, or they could be added as a result of unhygienic conditions of the baking environments, or because adequate precautionary measures are not taken to forestall cross-contaminations from other environmental sources. Several trace metals, at elevated levels, have been implicated in the aetiology of a myriad of ill health cases [30], [31]. For example, Cd causes renal dysfunction, obstructive lung disease, lung cancer, damage to human respiratory system. Lead leads to acute or chronic damage to the nervous system of humans and other behavioural disorders. Exposure to elevated levels of Zn can result in loss of appetite, decreased sense of taste and smell, slow wound healing, over exposure can lead to stomach cramps and skin irritations. High doses of Cu cause anaemia, liver and kidney damage, stomach and intestinal irritation. Associated to high levels of Fe are conjunctivitis, choroiditis, retinitis and so on Low-level Cr exposure can irritate the skin and cause ulceration while long term exposure can cause kidney or liver damage. Exposure to Ni causes lung cancer, respiratory failure, birth defects, heart disorders and asthma, among others. Aluminium at elevated levels can lead to loss of memory, severe trembling, damage to central nervous system. Manganese causes manganese poisoning, fatness, neurological symptoms, birth defects, while Co causes asthma, pneumonia, heart problems, thyroid damage, vomiting and nausea at elevated levels [32].

The aim of this study was to evaluate the levels of bromate and trace metals in the bread loaves sold within Ile-Ife and its environs. This would help to evaluate the extent to which the bread loaves conformed to safety guidelines with respect to their bromate and trace metal contents.

## 2 Experimental

### 2.1 Sampling

Nine widely consumed commercial bread samples were purchased from different outlets and bakeries within Ile-Ife and its environs. These samples were transported as bought to the laboratory for immediate preliminary treatment and subsequent analysis.

### 2.2 Sterilization of apparatus

All glassware and vials used were scrupulously cleaned by soaking overnight in a detergent solution in a wash basin. The glassware and vials were scrubbed clean with a nylon brush, rinsed with hot distilled water until no more soap was observed. They were then rinsed in cold distilled water, and soaked in 10% HNO_3_ for 48 h, rinsed properly with acetone and distilled water. They were finally oven-dried at 105 °C. Prior to use, the glassware and vials were stored in clean polythene bags that were securely sealed to prevent contamination by fallout from laboratory air.

### 2.3 Sample preparation and pre-treatment

Four slices of each bread samples were dried in an oven at 55 °C for 24 h. The crust formed was ground to fine powder using agate mortar and pestle. Accurately weighed 5 g powder of each sample was placed in a 250 mL beaker, and 2 × 10 mL distilled water was added and stirred thoroughly. The mixture was filtered into a 25 mL flask and was made up to the mark with distilled water.

### 2.4 Bromate determination

For this study, the method reported by Ojeka et al. [33] was adopted with slight modifications. With the aid of a calibrated pipette, 4 mL aliquot of each of the 9 bread samples was measured into 3 separate 25 mL calibrated flask. Added separately to this was 5 mL of 5 × 10^−4^ M solution of Congo Red or 5 mL of 5 × 10^−4^ M of Crystal Violet dye followed by 10 mL 2 M HCl solution. Dilution to 25 mL mark was made with distilled water, and gentle but thorough shaking was done prior to colorimetric analysis. Spectrophotometric measurements were made on a Jenway 6051 Colorimeter at *λ*_max_ = 580 nm for samples containing Crystal Violet and *λ*_max_ = 520 nm for samples containing Congo Red. All measurements were made at room temperature against distilled water as reference. The oxidation of the dyes by bromate was carried out in a hydrochloric acid medium:

(1) BrO_3_^−^ + 6H^+^ + 6e ⇌ Br^−^ + 3H_2_O

With irreversible oxidation indicators, the quantity of bromate solution consumes by the dyestuff indicator is exceedingly small and the indicator is bleached in the presence of 2 M HCl:

(2) 10Cl^−^ + 2BrO_3_^−^ + 12HCl ⇌ 11Cl_2_ + Br_2_ + 6H_2_O + 12e

The red colour of Congo Red changed to blue. Crystal Violet was purple in weak acid solution, green in strong acid solution and finally yellow. Both dyes were water soluble because of the low sulphuric acid groups (—SO_3_H) in Congo Red and dimethylamino groups in Crystal Violet.

### 2.5 Sample digestion

Accurately weighed 1.0 g of the solid powdered sample of each of the nine bread samples was weighed into a 100 mL Teflon beaker, 10 mL of concentrated HNO_3_ was added and the mixture was mixed gently and placed on a thermostated heating mantle maintained at 120–150 °C for about 1 h. Thereafter, 2 mL of HClO_4_ was added to the mixture and digested further for about 30 min. The mixture was removed from the heating mantle and the digested sample was quantitatively transferred into a 25 mL volumetric flask and filled to the mark with doubly distilled water in readiness for Atomic Absorption Spectroscopy analysis.

### 2.6 Trace metal quantification

The digested sample solution was used for Flame Atomic Absorption Spectrophotometer (FAAS) (FAAS, Buck Model 205) trace metal determination.

### 2.7 Quality control measures adopted

#### 2.7.1 Blank determination

Blank determination was carried out to ascertain the background levels of the analytes of interest in the materials and reagents used for analysis. This was done by running a separate determination under the same experimental conditions employed in the actual analysis of the sample, but excluding the sample. For the bromate determination, the absorbance by a dyesolution in an acidic medium was measured on the colorimeter. The corresponding concentration values obtained were subtracted from the values obtained for the bread samples. In the case of trace metals, blank determination was done by subjecting the same proportion of all the reagents used in the bread digestion to the same digestion protocol in the absence of bread samples. The values obtained from running blank determinations were subtracted from the analyte values as applicable.

#### 2.7.2 Calibration of FAAS

Although FAAS offers potential advantages such as simplicity, ruggedness, low cost and ease of miniaturization, its calibration was necessary to evaluate the response of the analytical procedure with respect to known quantities of the standards of the trace metals of interest so that the response to unknown quantities in the samples could be reliably estimated. For the FAAS 20, 18, 15, 12, 10, 7.5, 5, 3, and 1 μg/mL concentrations of each metal solution were freshly prepared by serial dilution for the determination of metals in the samples. These solutions were run on the FAAS to obtain the working calibration graph which was used to estimate the levels of trace metals in the samples by automatic interpolation with respect to the calibration graph.

#### 2.7.3 Calibration of colorimeter

The calibration curves for Crystal Violet dye method (at *λ*_max_ = 580 nm) and Congo Red dye method (at *λ*_max_ = 520 nm) were obtained by running the serially diluted bromate concentrations 0, 10, 20, 30, 40, 50, 60, 70, 80, 90 and 100 ppm, in accordance to the experimental conditions. The absorbance was plotted against the concentration values. The working graph obtained was used to extrapolate the bromate levels in a bread sample for both Crystal Violet and Congo Red dyes. The resultant experimentally determined relationships obtained are as contained in Eqs. (3), (4) for Crystal Violet and Congo Red respectively:

(3) $$y=0.003x+0.600\;(r^{2}=0.987)$$

(4) $$y=0.001x+1.396\;(r^{2}=0.978)$$

where *x* = concentration of bromate, and *y* = absorbance.

#### 2.7.4 Recovery experiment for trace metals

The method described by Oyekunle et al. [34] was used for recovery analysis. Two 1.0 g portions of bread samples from the same source were used for recovery analysis. One portion (*A*) was spiked with 25 μg/mL standard mixture of the trace metal solution while the other (control) portion (*A*′) was left unspiked. The two portions were separately but similarly taken through the procedures outlined earlier for sample digestion. The resulting solutions were subjected to FAAS analysis. The percentage recoveries (%*R*) of trace metals were determined by comparing the concentration values of each metal from the spiked and the unspiked sample results using the relationship:

(5) $$\%R=\frac{A-A^{\prime }}{B}\times 100$$

where *A* = trace metal concentration in spiked bread sample, *A*′ = trace metal concentration in unspiked bread sample and *B* = the amount of trace metal used for spiking.

#### 2.7.5 Recovery experiment for bromate

Two 5 g portions from the same bread sample, labelled A and B, were used for recovery analysis. One portion (A) was spiked with 4 mL of known concentration of bromate and the other sample (B) was left without external bromate addition. The two samples were subjected to bromate analysis as earlier outlined. The percentage recovery (%R) of bromate was triplicately determined by comparing the concentration values of bromate from the spiked and the unspiked sample results using the relationship:

(6) $$\%R=\frac{A-B}{C}\times 100$$

where *A* = bromate concentration in spiked bread sample, *B* = bromate concentration in unspiked bread sample and *C* = the amount of bromate used for spiking.

## 3 Results and discussion

### 3.1 Levels of bromate in bread samples

Table 1 gives the description and specification of the bread loaves used for the study. In all, there were nine different samples comprising of three big sliced, three big unsliced, and three small unsliced bread loaves considered in this study. All of them were specified as bromate free by the manufacturers. The respective standard calibration curve values (*r*^2^) obtained under the experimental conditions used in Table 2 were 0.987 and 0.978 for Crystal Violet and Congo Red methods, respectively. Thus, there were high linearity levels with which values of bromate in bread samples could reliably be compared for the two methods. However, the recovery analysis values of 50.24 ± 5.11% and 89.93 ± 2.46% obtained for Crystal Violet and Congo Red methods respectively indicated that the Congo Red method had a higher sensitivity than the Crystal Violet method thus making Congo Red method the preferred option to be relied upon in interpreting the bromate data obtained in the present study.

**Table 1:** Description of bread loaves used for analysis.

Code	Loaf size^a^	Loaf type	Manufacturer's specification
A	Big	Sliced	Bromate free
B	Big	Sliced	Bromate free
C	Big	Sliced	Bromate free
D	Big	Unsliced	Bromate free
E	Big	Unsliced	Bromate free
F	Big	Unsliced	Bromate free
G	Small	Unsliced	Bromate free
H	Small	Unsliced	Bromate free
I	Small	Unsliced	Bromate free

a Loaf size: Small ≈ 15 cm long; Big ≈ 30 cm long.

**Table 2:** Calibration curve (*r*^2^) and percentage recovery (%*R*) of bromate using the two methods.

Analytical method	Slope (m)	*r*^2^	%*R*
Crystal Violet method	0.003	0.987	50.24 ± 5.11
Congo Red method	0.001	0.978	89.93 ± 2.46

Based on the Congo Red method, the bromate levels (μg/g) in the bread samples (Table 3) ranged between 10.029 ± 0.007 in sample B and 66.224 ± 0.014 in sample C and occurred in the order B (10.029 ± 0.007) < E (21.397 ± 0.017) < D (22.356 ± 0.008) < H (23.326 ± 0.011) < F (24.461 ± 0.004) < G (26.258 ± 0.043) < A (36.012 ± 0.007) < I (40.231 ± 0.012) < C (66. 224 ± 0.014). These values were higher than the 0.55–0.62 or 0.10–12.10 or 1.16–6.68 μg/g bromate levels in bread respectively obtained by Sánchez et al. [35], Ojeka et al. [33] and Emeje et al. [36], but agreed closely with the 11.09–67.45 μg/g bromate levels in bread obtained by Abdulla and Hassan [37]. Since the Joint FAO/WHO [11] committee's recommendation of acceptance level of 0–60 mg KBrO_3_ bread was withdrawn because of long term toxicity and carcinogenicity studies *in vitro* and *in vivo* which revealed renal cell tumours in hamsters, it could be inferred that with respect to bromate content, none of the bread analyzed conformed to the zero level of bromate recommended as safety level for long term regular consumption of such bread. The bread samples A, C and I particularly showed much higher levels of bromate than recommended by NAFDAC [38] which implied that those bread samples could be hazardous for human consumption. The remaining bread samples showed considerably low levels of bromate in them. This, however, does not guarantee excellent consumption safety of the samples in view of possible long term toxic and carcinogenic effects that may emanate later. Sample C particularly had bromate level which was above the 0–60 μg/g level recommended by joint FAO/WHO. In general, none of the bread samples was actually bromate free as specified by the bakers.

**Table 3:** Bromate levels (μg/g)^a^ in the bread samples.

Sample code	Manufacturer's specification	[BrO_3_^−^] by Crystal Violet oxidation	[BrO_3_^−^] by Congo Red oxidation
A	Bromate free	28.025 ± 0.005	36.012 ± 0.007
B	Bromate free	3.129 ± 0.024	10.029 ± 0.007
C	Bromate free	41.336 ± 0.009	66.224 ± 0.014
D	Bromate free	2.051 ± 0.011	22.356 ± 0.008
E	Bromate free	7.667 ± 0.012	21.397 ± 0.017
F	Bromate free	4.205 ± 0.012	24.461 ± 0.004
G	Bromate free	10.313 ± 0.012	26.258 ± 0.043
H	Bromate free	6.333 ± 0.023	23.326 ± 0.011
I	Bromate free	20.296 ± 0.022	40.231 ± 0.012

Bromate specified level: 0 μg/g [38]; 0–60 μg/g [11].

a Value = mean of triplicate determinations ± s.d.

### 3.2 Levels of trace metals in bread samples

The reliability of the analytical procedures adopted in this study was tested in terms of sensitivity, recovery, precision and accuracy. Table 4 shows the calibration curve values (*r*^2^) and percentage recovery (%*R*) for the heavy metals as determined experimentally using FAAS. Under the experimental conditions used, the standard calibration curves obtained showed high linearity level with correlation values (*r*^2^) ranging between 0.9752 and 0.9956. Recoveries of heavy metals ranged from 78.92 ± 4.15% in Co to 95.91 ± 3.56% in Zn. These values were adjudged acceptable. The percentage relative standard deviation (%RSD) values obtained for bread samples (2.76–5.23% RSD) showed that precision was better than 10% RSD level.

**Table 4:** Calibration curve (*r*^2^) and percentage recovery (%*R*) of metals in bread sample.

Trace metal	Calibration curve, *r*^2^	(%*R*)
Cu	0.9834	90.25 ± 3.65
Co	0.9956	78.92 ± 4.15
Mn	0.9696	85.35 ± 2.76
Pb	0.9919	93.22 ± 5.23
Zn	0.9752	95.91 ± 3.56

Trace metals content in the various bread samples are listed in Table 5. Levels of Co ranged between 0.03 ± 0.01 and 0.10 ± 0.03 μg/g. Cobalt is essential to life in only minute amounts. Food and Agricultural Organization (FAO) and World Health Organization (WHO) recommend a 2.4 μg/day of vitamin B_12_ which is equivalent to 0.1 μg/day of cobalt as safety level in adult diet. For most of the bread samples analyzed, Co levels fell around this value. However, considering the amount of bread a regular consumer may take up per day, the levels of Co that may come from bread alone appeared to far exceed the level recommended by joint FAO/WHO [11] which implies that regular eaters of bread may suffer harmful effects overtime. Cobalt is the active centre of coenzymes called Cobalamins; the most common is vitamin B_12_. As such, it is an essential trace dietary mineral for all animals. Cobalt in inorganic form is also an active nutrient for bacteria, algae and fungi. The minimum presence of cobalt in soils therefore markedly improves the health of grazing animals and an uptake of 0.20 mg/kg a day is recommended as they can obtain vitamin B_12_ in no other way. The LD_50_ value for soluble cobalt salts has been estimated to be between 150 and 500 mg/kg [11].

**Table 5:** Trace metal levels^a^ in the analyzed bread samples (μg/g).

Code	Bread type	Co	Cu	Mn	Pb	Zn
A	Sliced	0.08 ± 0.01	0.38 ± 0.03	75.53 ± 1.02	0.08 ± 0.01	6.63 ± 0.25
B	Sliced	0.09 ± 0.02	0.30 ± 0.06	73.90 ± 0.56	0.05 ± 0.01	4.98 ± 0.12
C	Sliced	0.06 ± 0.02	0.38 ± 0.03	72.23 ± 1.21	0.09 ± 0.03	5.04 ± 0.31
D	Unsliced	0.08 ± 0.01	0.46 ± 0.12	43.90 ± 1.06	0.06 ± 0.02	5.38 ± 0.11
E	Unsliced	0.10 ± 0.03	0.35 ± 0.04	64.10 ± 3.11	0.10 ± 0.02	4.99 ± 0.12
F	Unsliced	0.07 ± 0.01	0.35 ± 0.05	52.75 ± 0.29	0.07 ± 0.02	3.84 ± 0.04
G	Unsliced	0.08 ± 0.02	0.38 ± 0.02	49.68 ± 2.13	0.09 ± 0.03	3.88 ± 0.08
H	Unsliced	0.09 ± 0.02	0.23 ± 0.06	47.40 ± 1.35	0.06 ± 0.02	5.03 ± 0.03
I	Unsliced	0.03 ± 0.01	0.39 ± 0.05	25.83 ± 0.59	0.03 ± 0.00	2.23 ± 0.16

Mean ± s.d.		0.08 ± 0.02	0.36 ± 0.05	56.15 ± 1.26	0.07 ± 0.02	4.67 ± 0.14
CV		25.0	13.9	2.24	28.6	3.0

a Value = mean of triplicate determination ± standard deviation.

The level of Cu in the bread samples used for this study was in the range of 0.23 ± 0.06 to 0.46 ± 0.12 μg/g. Copper is the strongest pro-oxidant for oils, and for the best stability, the content of Cuin fat and oil or their products should be below 0.02 μg/g [39], [40]. The amount of Cu detected in the samples was more than ten folds higher than 0.02 μg/g. This implies that rapid deterioration of the vegetable oil used as releasing agent in baking of bread would be inevitable. In addition to the amount that came from the cereal raw materials used for flour production, the contamination of Cu may also be due to the degradation and deterioration of some copper alloy equipment used during flour making and bread baking. Like Co, Cu is an essential mineral that is found throughout the body; it helps the body in making red blood cells and keeps nerve cells and the immune system healthy. It also helps in forming collagen, a key part of bones and connective tissue [41], [42]. At a suitable level in human system, Cu may also act as an antioxidant, getting rid of free radicals that can damage cells and deoxyribonucleic acid (DNA). Copper deficiencies are rare, as the human body consistently stores copper and requires very little copper in order to function effectively. However, there are certain groups of individuals that live with a higher risk [42]. Individuals who are required to consume large amounts of zinc, vitamin C, or fructose may be at risk of Cu deficiencies as these minerals tend to deplete Cu levels. Additionally, patients who have conditions that cause low rates of absorption may not be able to retain the needed trace amounts of Cu their bodies require. Recent studies have shown that infants who are exclusively fed cow milk formula may acquire Cu deficiencies as the milk itself is low in Cu [43]. Similarly, premature infants, particularly those who are delivered with severely low birth weights are at a greater risk for developing Cu deficiencies. A number of nutrition surveys have indicated that the diets of approximately 25% of adolescents, adults, and people over 65, do not meet the recommended daily nutrient intake for Cu [43]. Fortunately, Cu deficiency can be confirmed by very low serum metal and ceruloplasmin concentrations in the blood. Other conditions previously linked to Cu deficiency include osteoporosis, osteoarthritis, rheumatoid arthritis, cardiovascular disease, colon cancer, and chronic conditions involving bone, connective tissue, heart, and blood vessels. Copper helps the body absorb iron, and the human body needs Cu to make energy [43]. The 0.23 ± 0.06 to 0.46 ± 0.12 μg/g Cu levels detected in the samples were adequate for daily needs of humans because the values are within the 0.008–9 μg/g levels set for Cu levels in foodstuffs [44], [45].

Manganese (Mn) levels in the bread samples ranged from 25.83 ± 0.59 to 75.53 ± 1.02 μg/g. Manganese, a trace mineral that participates in many enzyme systems in the body, is found widely in nature, but occurs only in trace amounts in human tissues and was first considered an essential nutrient in 1931 [46]. It plays a role in bone mineralization, protein and energy metabolism, metabolic regulation, cellular protection from damaging free radical species, and the formation of glycosaminoglycans [46]. A diet deficient in Mn could lead to poor growth and impaired reproduction. The human body contains a total of 15–20 mg of Mn, most of which is located in the bones, with the remainder found in the kidneys, liver, pancreas, pituitary glands, and adrenal glands. Although Mn is an essential nutrient, excess Mn affects the CNS and neurological effects have been observed in case of occupational exposure [46]. The main contributors of Mn to the diet are cereals (10–30 mg/kg), vegetables and fruits (0.5–5 mg/kg) while nuts may have a higher content [47], [48]. The levels of Mn found in the bread samples in this study far exceeded the daily intake of 2–3 mg/day recommended by WHO [49] implying that the bread samples may contribute to the ill effects excess Mn in meal may cause to consumers overtime.

The lead (Pb) content of the bread was in the range of 0.03 ± 0.00 to 0.10 ± 0.02 μg/g. Lead is a highly poisonous metal affecting almost every organ and system in the body. The FAO/WHO Joint Expert Committee on Food Additives [49] has established a Provisionally Tolerable Weekly Intake (PTWI) at 0.025 mg/kg body weight. The Pb content of the bread was clearly greater than the PTWI level for human intake. This could be a source of future health problems. The widespread usage of Pb for thousands of years has resulted in its increased concentrations in the environment. Lead when absorbed into the body can be deposited for a long period of time in some tissues where it is later released into the bloodstream and distributed within the body. Principally, Pb in the body affects the nervous system, kidneys and blood. Lead exposure is a possible source of tumours and United States Environmental Protection Agency (USEPA) has classified Pb as a Group B2 carcinogen *i.e.* a probable human carcinogen (USEPA http://www.epa.gov/ttn/atw/hlthef/hapindex.html) [50]. Lead exposure also causes small increase in blood pressure (Codex Committee on Food Additives and Contaminants [51]).

In the present study, the levels of Zn in bread samples were in the range of 2.23 ± 0.16 to 6.63 ± 0.25 μg/g. Zinc is one of the most ubiquitous of the essential trace metals [52]. Zinc aids the function of a large number of metallo-enzymes [46], [47]; supports normal growth and development during pregnancy, childhood, and adolescence and is required for proper sense of taste and smell [41]. A daily intake of Zn is required to maintain a steady state because the body has no specialized Zn storage system. Zinc also diminishes the toxicity of Cd and Cu [52], but its deficiency or excessively high levels may enhance susceptibility to carcinogenesis [47]. Zinc deficiency is characterized by growth retardation, loss of appetite, and impaired immune function. In more severe cases, zinc deficiency causes hair loss, diarrhoea, delayed sexual maturation, impotence, hypogonadism in males, and eye and skin lesions, weight loss, delayed healing of wounds, taste abnormalities, and mental lethargy [30], [53]. FAO/WHOJECFA [54] established a Provisional Maximum Tolerable Daily Intake (PMTDI) of 1 mg/kg body weight. In 1982, JECFA proposed a daily dietary requirement of zinc of 0.3 mg/kg of body weight and a provisional maximum tolerable daily intake (PMTDI) of 1.0 mg/kg of body weight [54]. The daily requirement for adult humans is 15–22 mg/day [55]. The levels of Zn in the bread samples (ranging from 2.23 ± 0.16 to 6.63 ± 0.25 μg/g) fell below the WHO recommended level of consumption for humans. Thus a regular consumer of bread in this environment might suffer associated zinc-deficiency diseases.

## 4 Conclusion

The study evaluated the bromate and trace metal levels of bread commonly sold and consumed in Ile-Ife and its environs. It was revealed that the millers and bakers did not comply with the bromate free rule stipulated by both NAFDAC and WHO contrary to the “bromate free” indicated on their labels. In addition, the bromate levels in some of the breads were higher than the 60 μg/g stipulated by the WHO. Results of trace metals analysis showed that bread samples contained Co, Cu, Mn, Pb and Zn at levels that were at wide variance with those specified by such bodies as NAFDAC or WHO. Particularly, the Pb content of the bread was greater than the 0.025 mg/kg body weight set as the Provisionally Tolerable Weekly Intake for humans. There is the need to place a close surveillance on bakers of bread in order to ensure better compliance with health regulations.

## Transparency document

Transparency document
